# Prevalence, nature, and determinants of COVID-19-related conspiracy theories among healthcare workers: a scoping review

**DOI:** 10.1192/j.eurpsy.2025.12

**Published:** 2025-03-03

**Authors:** Hanne Loyens, Johan Detraux, Marc De Hert

**Affiliations:** 1 University Psychiatric Center, Katholieke Universiteit Leuven, Leuven, Belgium; 2 University Psychiatric Center, Katholieke Universiteit Leuven, Kortenberg, Belgium; 3 Department of Neurosciences, Center for Clinical Psychiatry, Katholieke Universiteit Leuven, Leuven, Belgium; 4 Leuven Brain Institute, Katholieke Universiteit Leuven, Leuven, Belgium; 5 Antwerp Health Law and Ethics Chair, University of Antwerp, Antwerp, Belgium

**Keywords:** conspiracy theory, COVID-19, healthcare workers, prevalence

## Abstract

**Background:**

COVID-19-related conspiracy theories (CTs) have been observed among healthcare workers (HCWs). There exists, however, a lack of research investigating the extent, nature, and determinants of CTs among HCWs worldwide.

**Methods:**

A systematic literature search of Medline, EMBASE, Web of Science, Scopus, and CINAHL electronic databases (from inception to October 2023) was conducted for studies examining the prevalence and nature of COVID-19-related CTs among HCWs and health students and/or factors driving HCWs into believing these CTs.

**Results:**

Prevalence rates of COVID-19-related CTs among HCWs varied widely across studies, ranging from 0.89% to 75.6%. These prevalence rates mainly concern vaccine-hesitant HCWs (although a minority of vaccinated HCWs also endorse CTs). Higher prevalence rates of CTs were found in the Arab world, Ethiopia, and Nigeria, compared to other African and Western countries. While in European countries and Northern America, an increased belief of HCWs in the “destabilization and power gain” narrative was found, African HCWs particularly endorsed the “population reduction” and “liberty restriction” narratives. Limited and heterogeneous data prevented conclusive findings on the relationship between CTs and sociodemographic factors, ethnicity, and psychological traits among HCWs. However, a consistent observation emerged regarding the level of education, indicating HCWs with higher educational attainment (e.g., physicians) tend to endorse CTs less frequently.

**Conclusion:**

Although COVID-19-related CTs may be highly prevalent among vaccine-hesitant HCWs, gaps in understanding the drivers of CTs among HCWs remain. Given HCWs’ critical role in public health, especially during pandemics, further research is therefore essential.

## Background

In 2019, the World Health Organization (WHO) identified vaccine hesitancy, defined as the “*delay in acceptance or refusal of vaccination despite the availability of vaccine services*” [1], as one of the 10 threats to global health [2]. Although there have always been people hesitant towards receiving vaccinations, this threat has only increased since the beginning of the COVID-19 pandemic [1, 3–6]. For example, a dramatic decrease in the administration of measles-containing vaccines, especially in children older than 24 months, was observed from March 16, 2020 to April 19, 2020 [6] The rapidity of the COVID-19 vaccine development and concerns regarding the vaccine’s safety certainly have contributed to the lack of vaccine confidence [7, 8]

Several factors have been found to be associated with vaccine hesitancy towards the COVID-19 vaccine, such as sociodemographic (e.g., education), health-related (e.g., vaccination history/medical conditions), and vaccine-related (e.g., concerns about the safety or quality of the vaccine) factors [9]. However, conspiracy theories (CTs) are another important factor associated with vaccine hesitancy. Moreover, CTs even have been identified as the strongest predictor of anti-vaccination attitudes [10].

CTs can be defined as secret plans hatched by powerful groups (“elites”) with the intention to harm society or a specific group of people, often to the benefit of the powerful group [11–13]. While many CTs are unjustified or irrational beliefs, as they have little or no evidence [14], some CTs may become plausible for people with a deep-rooted mistrust of government, medicine, and/or science, caused by countless historical examples of abuse or historical marginalization, or for people within certain socio-economic or political situations, such as a lack of economic vitality and undemocratic regimes [10].

Despite their scientific and medical training, healthcare workers (HCWs) and healthcare students have been identified as a sub-group displaying considerable hesitancy towards accepting a COVID-19 vaccine [7, 15, 16]. Although the prevalence of COVID-19 vaccination hesitancy in HCWs varied widely, a large-scale review published in 2021 found that among HCWs (*n* = 76,471) more than a fifth of HCWs worldwide reported COVID-19 vaccination hesitancy [16]. The vaccine hesitancy rate among healthcare students is almost equal to the hesitancy rate in practicing HCWs [15]. Limited information, however, exists about the prevalence and determinants of COVID-19-related CTs in HCWs and healthcare students worldwide. The purpose of this study therefore was to conduct a scoping review to map out the evidence base pertaining to (1) the prevalence of COVID-19-related CTs among HCWs and healthcare students worldwide, and (2) the nature and determinants of conspiracy thinking among HCWs within the context of the COVID-19 pandemic. Getting insight into the factors contributing to these beliefs among this population is pivotal as HCWs COVID-19 vaccine hesitancy has numerous consequences that negatively affect coworkers, patients, and the healthcare system [17].CTs held by these people may foster (more) distrust towards health authorities and their recommendations, which could impede efforts to end pandemics [18].

## Methods

### Search strategy

A comprehensive and systematic literature search of Medline, EMBASE, Web of Science Core Collection, Scopus, and CINAHL electronic databases (from inception to October 2023) was conducted for English, Dutch, and German studies, examining the prevalence of COVID-19-related CTs among HCWs and healthcare students, and/or factors driving HCWs into believing these theories. Full search strategies are available as Supplementary Material. Duplicates were removed by J.D., using EndNote X9. After removing duplicates, titles and abstracts were screened by H.L., using Rayyan QCRI. H.L. and J.D. did the full-text screening. Articles that were deemed potentially relevant according to the selection criteria were included. Any disagreements were solved by consensus or by the decision of a third reviewer (M.D.H.). References of the identified studies and pertinent reviews were carefully cross-checked for additional relevant studies.

### Eligibility criteria

Studies were eligible for inclusion if they:

1. were peer-reviewed;
2. reported prevalence rates of COVID-19-related CTs and/or explored the determinants of these CTs;
3. labelled CTs as beliefs featuring a secret plot by a group of powerful elites that involve the harm of a group of people [11, 13];
4. were conducted at a time when vaccines were available in the studied country or region;
5. included a population of HCWs and/or healthcare students. For defining HCWs, we used the International Standard Classification of Occupations (ISCO), also used by the WHO [19]. This classification includes health professionals (e.g., generalist medical doctors, nursing professionals, midwifery professionals, dentists, pharmacists, physiotherapists, dieticians, and nutritionists), health associate professionals (e.g., technicians for medical imaging, laboratory work, and dental prosthetics, pharmaceutical and dental assistants, community health workers, ambulance workers), personal care workers in health services (e.g., healthcare assistants, home-based personal care workers), health management and support personnel (e.g., health service managers, biomedical engineers, medical secretaries) and other health service providers.

Studies that were not peer-reviewed or published (preprints, dissertations, conference papers, books/book sections, commentary/opinion pieces), studies exclusively presenting qualitative data, case reports, and non-original research were excluded. Studies including other professions not covered by the WHO definition of HCWs (e.g., studies with first responders that also include enforcement officers and firefighters, next to HCWs, without providing separate data for HCWs), as well as studies written in other languages than English, Dutch or German were excluded. When conspiracy beliefs were not embedded into a belief system involving a secret plot, the study was also excluded.

### Data extraction

Data were extracted and mapped descriptively by H.L., using a data extraction form. This form included the following information: author(s), year of publication, country/region where the study has been conducted, study design, specific population of HCWs and/or healthcare students, sample size, mean age, gender, ethnicity, vaccine hesitancy rate(s) due to CTs, and/or information on the determinants or nature of CTs. We refrained from employing meta-analytical methods due to the significant heterogeneity of the included studies regarding methodology, measures, and outcomes.

## Results

### Search strategy

**Figure 1.**
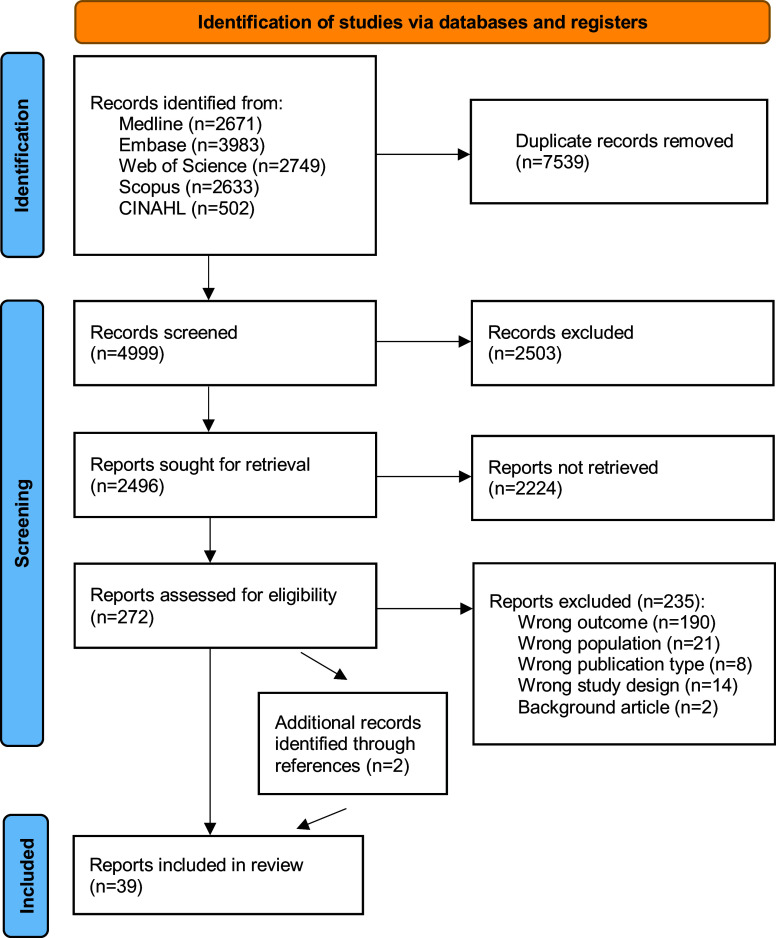
PRISMA flow chart.

The original search in the Medline, EMBASE, Web of Science, Scopus, and CINAHL databases yielded a total of 12,538 reports (Medline: 2,671; Embase: 3,983; Web of Science: 2,749; Scopus: 2,633; CINAHL: 502). Of these, 7,539 duplicate reports were removed (see Figure 1). Overall, 272 references of published reports were selected as potentially eligible, of which 37 reports met the inclusion criteria. Two published reports, identified through cross-reference, were added (see Figure 1) [12, 20–57].

### Study and patient characteristics

The 39 eligible reports included 37 studies with a total of 55,556 participants. Roberts [42] and Dubov [43] extracted their data from Dubov [44] for secondary analysis. These reports therefore were counted as one study. All studies were performed between 2021 and 2023. Most studies were conducted in the Arab world (*n* = 10). The other studies were conducted in Africa (not belonging to the Arab world) (*n* = 9), Asian countries (*n* = 3), or European countries (*n* = 6), Turkey (*n* = 4), and North America (*n* = 3). Two studies were conducted worldwide (*n* = 2). Of the 37 eligible studies, 33 had a cross-sectional design, 1 was a prospective cohort study, and 3 were mixed-method studies. Mean age was 32.8 years (SD = 6, range: 18–78); 58.0% of the participants were female. All patient and study characteristics of the included studies are presented in Table 1.

**Table 1.** Characteristics of quantitative studies, including conspiracy findings and/or correlation between different determinants and CTs among HCWs and healthcare students

Reference	Country	Study design	Healthcare workers	*N*	Mean age in years (±SD)	Female (%)	Race/ ethnicity (%)	COVID-19 vaccination status and data collection	Prevalence of CTs and/or correlation between different determinants and CTs among HCWs and students
Azimi et al. [20]	Afghanistan	Cross-sectional	Medical students in clinical years (4th, 5th, 6th, and 7th year) of five universities	459	21.00 (±NR)	70.30%	NR	Almost half of the participants (51.5%) were already vaccinated; 42.3% vaccine hesitancy DC: March-June 2022	**“There is a chip in the vaccine”: *n*** = **4 (0.89%)**
Joseph et al. [21]	Sierra Leone	Cross-sectional	Clinical and non-clinical staff in six facilities (hospital, health center…)	609	NR	45.35%	NR	49.8% fully or partially vaccinated; 50.2% not yet vaccinated. DC: March-May 2022	**“Vaccine designed to harm me, e.g., conspiracy”: *n*** = **23 (3.8%)** **Clinical staff: 3%** **Non-clinical staff: 4%**
Oyeyemi et al. [22]	Nigeria	Cross-sectional	Medical doctors, nurses, pharmacists, laboratory scientists, community health extension officers or workers, health assistants and others	557	NR	71.70%	NR	75.2% vaccine acceptance; 23.5% vaccine hesitancy DC: March-May 2021	*“I think COVID–19 vaccine is a means to implant digital microchips to track and control people”: n* = *147 (26.7%)* *[aOR] men vs. women (95% CI): 1.4 (0.8–2.5)* *[OR] low level of trust in government (95%CI): 4.6 (2.6–8.0)* *[aOR] nurses vs. physicians (95% CI): 3.9 (1.3–12.0)* *[aOR] pharmacists vs. physicians (95% CI): 3.0 (0.4–22.0)* *[aOR] laboratory scientists vs. physicians (95% CI): 5.1 (1.0–25.9)* *[aOR] CHEO vs. physicians (95% CI): 4.0 (1.2–13.8)* *[aOR] health authority as main source (vs media) (95% CI): 0.4 (0.2–0.7)* *“I think COVID–19 vaccine will alter my DNA or genetic information” : n* = *167 (30.5%)* *[aOR] men vs. women (CI 95%): 1.8 (1.1–3.2)* *[OR] low level of trust in government (95%CI): 5.2 (3.1–8.8)* *[aOR] nurses vs. physicians (95% CI): 2.2 (0.9–5.4)* *[aOR] pharmacists vs. physicians (95% CI): 3.1 (0.6–16.2)* *aOR] laboratory scientists vs. physicians (95% CI): 1.9 (0.4–7.9)* *[aOR] CHEO vs. physicians (95% CI): 1.7 (0.6–4.5)* *[aOR] health authority as main source and belief in CTs (vs media) (95% CI): 0.5 (0.3–0.9)*
Akova et al. [23]	Turkey	Cross-sectional	Physicians, nurses/midwives and others	1111	34.3 (±9.2)	59.6%	NR	NR DC: February-March 2022	*“The virus is man-made and part of a conspiracy plan”: n=516 (46.4%)*
Bereda et al. [24]	Ethiopia	Cross-sectional	HCWs working in a registered healthcare setting (physician, midwive, nurse, health officer, laboratory technician and others)	422	NR	45.5%	NR	69.7% vaccine hesitant DC: June 2021 – June 2022	*“Belief in CTs”: n=319 (75.6 %)* [aOR] Belief in CTs and vaccine hesitant vs. non-hesitant (95%CI): 2.43 (1.948–5.170)*
Almojaibel et al. [25]	Saudi Arabia	Cross-sectional	Physician, nurse, dentist, pharmacist, other health care specialists, technician in allied medical sciences	505	NR	NR	NR	74.5% vaccinated + 9.5% registered to receive it. 9.3% resistant or hesitant DC: April 2021	**“It has a chip to control me”, “It will change my DNA”: n=25 (5%)**
Kaya [12]	Turkey	Cross-sectional	HCWs at the hospital (midwife, nurse, technician, medical laboratory technique, research assistant)	128	30.97 (±8.07)	NR	NR	NR DC: February-March 2021	*Belief in CTs not correlated with age (NS)* Belief in CTs negatively associated with positive attitude towards vaccination** Research assistants, participants who had higher education attainments and those with a longer duration of working life: less likely to believe in CTs** HCWs with bachelor’s degrees and below: more likely to believe in CTs than HCWs with master’s and doctorate degrees*
Rezq et al. [26]	Jordan	Cross-sectional	Nurses at three private hospitals	189	30.2 (±3.7)	75.7%	NR	81% were vaccinated, 19% did not have a vaccine DC: July-August 2021	*“COVID–19 is man-made”: n=86 (45.5%)*
Satti et al. [27]	Sudan	Cross-sectional	Community pharmacists	382	30.4 (±5.6)	65.4%	NR	74.9% received or intend to receive a vaccine DC: July-September 2022	***“COVID–19 is a man-made virus and part of a conspiracy plan”: n=111 (29.1%)*** HCW with CT beliefs were still more likely to accept vaccination: 62.2%** [OR] Vaccine hesitancy and belief in CTs (95%CI): 0.44 (0.23–0.85)*
Fountoulakis et al.[28]	Worldwide (40 countries)	Cross-sectional	Doctors, nurses, administrative staff in hospitals, other healthcare profession and hospital staff	12,792	NR	62.40%	NR	NR DC: April 2020 - March 2021.	*“Belief in CTs”: approx. 33%* *“COVID–19 is the result of 5G antenna technology”: 20.81%* *“Believing in the deliberate inflation of death rates by government”: 44.24%* HCWs with current depressive symptoms: higher tendency in believing in CTs*
Vranic et al. [29]	Bosnia and Croatia	Cross-sectional	Medical students of UNSA University (Bosnia), UNIRI University (Croatia), and UNIRI-E University (where 85.3% of German students of medicine in English)	557	NR	NR	NR	98.8% to 100% were vaccinated. 3.6% to 30.2% of medical students had a negative or hesitant attitude against vaccination. DC: February-May 2021	*“The pharmaceutical industries are creating infections with the goal of increasing earnings”:* *UNSA: n* = *27 (16.0%)* *UNIRI: n* = *143 (42.9%)* *UNIRI-E: n* = *26 (46.4%)*
AlKhawaldeh et al. [30]	Jordan	Cross-sectional	HCWs in public, private, and university hospitals: (70.1%) nurses, doctors, pharmacists, respiratory therapists, lab technicians and nutritionist/dietitians	904	35.04 (±9.07)	53.80%	NR	Participants were not yet vaccinated. 51.7% were vaccine-resistant: 17.5% vaccine vaccine-acceptant. The rest was vaccine-hesitant. DC: January-February 2021	*“COVID–19 vaccination is a conspiracy”: n* = *228 (25.2%)*
Azizoğlu et al. [31]	Turkey	Cross-sectional	HCWs at a private hospital (nurses, technical, medical records and allied health personnel, physicians)	309	28.48 (±9.09)	NR	NR	74.8% were vaccinated DC: March-April 2021	*“I believe that they will inject microchips to people with the coronavirus vaccine”: n* = *7 (2.2%)* *“I believe that the coronavirus vaccine will be the end of humanity”: n* = *11 (3.6%)* *“I think the coronavirus vaccine is a complete fabrication”: n* = *23 (7.4%)*
Konje et al. [32]	Tanzania	Cross-sectional	Nurse, clinical officer, medical officer, and specialist in different health facilities (dispensary, health center, district hospital, regional hospital and tertiary hospital)	811	35 (±9.04)	48%	NR	18.5% were vaccinated DC: September 2021	***“Belief in CTs”: n*** = ***42 (5.2%)*** ***Correlation belief in CTs (3.5%) and vaccine willingness (1.7%) vs. vaccine hesitancy (3.5%) (NS)***
Demeke et al. [33]	Ethiopia	Cross-sectional	Medical doctors, nurses, pharmacy, midwifery, laboratory, anesthesia, physiotherapy, optometry and others	319	NR	25.1%	NR	45% received the vaccine; 54.2% did not. DC: May-June 2021	*“Being a plot or conspiracy”: n* = *96 (30.1%)*
Hoffman et al. [34]	USA	Mixed-Method	HCWs, health science students on Twitter	106	NR	NR	NR	93% received at least one dose of the vaccine DC: April-June 2021	*“Belief in CTs”: n* = *0 (0%)*
Odejinmi et al. [35]	United Kingdom	Mixed-method	Midwives employed in two teaching hospitals	378	NR	99%	White:66.93% Black:21.16% Asian: 3.44% Mixed Race: 5.03% Other: 2.38%	75% accepted the vaccine DC: NR	**“The government is able to track you”: *n*** = **13 (3%)** **[aOR] Belief in CTs Black vs. White (95%CI): 0.97 (0.24–3.84) (NS)**
Asres et al. [36]	Ethiopia	Cross-sectional	Students of medicine, medical laboratory, pharmacy, health officer, nursing, anesthesia, environmental health, midwifery	387	21.97 (±1.67)	44.9%	NR	27.1% vaccine acceptant; 45.7% vaccine hesitant DC: NR	**“It is a biological weapon”: *n*** = **68 (16.8 %)** **“It is a political game”: *n*** = **118 (30.5%)** **“Vaccination is a money-making venture”: *n*** = **9 (12.7%)**
Al-Qudah et al. [37]	Jordan	Cross-sectional	Healthcare specialties and healthcare students (applied health sciences, dentistry, medicine and surgery, nursing, pharmacy, other healthcare specialties)	1409	NR	NR	NR	NR DC: March 2021	*“COVID –19 is a political manipulation”: approx. 20%* *“The virus is bioengineered”: approx. 30%* *“Vaccines are manufactured to increase pharmaceuticals”: approx. 20%* *“COVID–19 pandemic aims to place a microchip in”: approx. 5%* Medical students and graduates: less CTs compared to other HCWs *(exception vs nurses NS)
Habib et al. [38]	Saudi Arabia	Cross-sectional	Medical students	1445	NR	11.3%	Saudi: 98.8% Non-Saudi: 1.2%	16% were not vaccinated DC: August-October 2021	**“The COVID–19 vaccine involves a conspiracy”: *n*** = **234 (48.6%)** **97.9% of students believing in CTs were preclinical students**
Jamil et al. [39]	Pakistan	Cross-sectional	Undergraduate medical students from different medical universities	401	NR	73.8%	NR	71.3% were vaccinated DC: June-August 2021	*“World superpowers use it as a cover to launch a vaccination program to facilitate a global surveillance regime and establish one world order”: n* = *153 (38.1%)* *“COVID–19 virus is a bioweapon released deliberately by the Chinese government to control the world’s population” n* = *106 (26.4%)* *“Pandemic is a hoax perpetrated by a global to diverge Muslim belief by shutting down mosques”: n* = *63 (15.7%)* *Correlation CT and gender (NS)* *Correlation CT and year of study (NS)* Correlation absence of belief in CTs and vaccinated HCWs*
Petersen et al. [40]	Germany	Cross-sectional	Nursing, administrative staff, medical-technical staff, physicians, and scientific staff in hospitals	1683	NR	78.7%	NR	NR DC: January-June 2021	CTs negatively associated with vaccination willigness.* Physicians and scientific staff: less CTs beliefs vs. nurses, medical-technical, and administrative staff.* Administrative and nursing staff: most CT beliefs.* Women: more CT beliefs vs. men (with small to very small differences)* *Correlation age and CTs (NS)*
Inah et al. [41]	Nigeria	Cross-sectional	Medical radiation workers (radiologists, radiographers, radiotherapists, medical physicists, and radiology nurses)	50	38.04 (± 12.25)	32%	NR	Willingness to receive vaccine: 45.45%; unwillingness: 54.55% DC: May 2021	**“The Western world plans to destroy the world”: 8.40%** **“Plans to systematically alter DNA signaling”: 10.69%** **“It has to do with 5G technology”: 5.3%**
Dubov et al. and Roberts et al. [42–44]	USA	Cross-sectional	Physicians, nurses, advanced practice providers, pharmacists, other allied health professionals, administrators and nonclinical ancillary staff at academic and private hospitals	2491	NR	74.95%	White: 72.8% Black/ African: 4.94% Asian:17.58% Pacific Island: 1.89% Native: 2.73%	84.4% were vaccinated DC: February-April 2021	***CTs among all HCWs:*** ***“The virus is or could be manmade”: n*** = ***947 (38%)*** [aOR] unvaccinated HCWs with “manmade -belief” vs. non-belief (95% CI): 1.37 (1.12–1.68)* ***Hispanic: 22.98%*** ***African-American: 20.33%*** ***Asian American: 13.47%*** ***“The pandemic is a hoax”: n*** = ***149 (6%)*** ***[aOR] unvaccinated HCWs with “hoax -belief” vs. non-belief (95%CI): 0.82 (0.62–1.10) (NS)*** ***“The pandemic is a hoax”:*** ***Hispanic: 3.68%*** ***African-American: 1.63%*** ***Asian American: 3.42%*** ***“Misinformed HCW group” (n*** = ***38): up to 92% believed CTs. They were slightly older, leaned Republican, and came from all levels of education.*** ***“Unconcerned HCW group” (n*** = ***86): up to 13% believed CTs. They were younger, racially diverse, most educated, and leaned Democrat.*** ***CT among nurses:*** ***“COVID–19 is a fabrication or a hoax, a synthetic virus manufactured under nefarious motives such as bioterrorism, economic destabilization, population control”: n*** = ***212 (24 %)*** ***Vaccine acceptance nurses:*** ***(willing to be) vaccinated who believe in conspiracy: 19.3%*** ***Unwilling/not vaccinated who believe in conspiracy: 43%*** ***[OR] belief in CTs and vaccine acceptance vs. non-belief (95%CI): 2.05 (1.29–3.25)*** ***Vaccine acceptance HCW of color:*** ***[aOR] lower acceptance of CTs vs higher acceptance with CT belief (95%CI): 1.39 (1.10–1.76)***
Nasr et al. [45]	Lebanon	Cross- sectional	Dentists	529	40.54 (±14.01)	44.80%	NR	Vaccination acceptance rate 86%. DC: February 2021	*“I believe that COVID–19-vaccination is a conspiracy”: (apr. 5%)*
Szmyd et al. [47]	Poland	Cross-sectional	Physicians and administrative healthcare assistants	387	NR	68.50%	NR	94.44% of doctors planning to get vaccinated (assistants: 61.48%), 5.56% of doctors not planning to get vaccinated (assistants:38.52%) DC: December 2020-January 2021	*“Belief in CTs (overall)”: n* = *30 (7.75%)* *Physician: 3.17%* *Healthcare assistant: 16.3%* *“Microchip injection”: n* = *5 (1.29%)* *Physician: 0%* *Healthcare assistant: 3.7%* *“Control of births by vaccine manufacturers”: n* = *12 (3.10%)*
Ditekemena et al. [48]	Democratic Republic of Congo	Cross-sectional	HCWs	324	NR	NR	NR	55.9% willing to be vaccinated; 44.1% not willing to be vaccinated 149 HCWs were vaccine hesitant DC: August-September 2020	**“They want to kill us”: *n*** = **10 (6.6%)** **“They want to make us sterile”: *n*** = **5 (3.3%)** **“There are several CTs going around”: *n*** = **1 (7.1%)**
Woolf et al. [49]	United Kingdom	Prospective cohort study	All HCWs or ancillary workers	11,584	45 (±*N*R)	75.9%	White: 70.3%; Asian: 19.2%; Black: 4.2%; Other 6.4%	23.3% vaccine hesitant DC: February 2021	Higher COVID–19 CBS-score with vaccine hesitant HCWs*** [OR]: CBS-score with vaccine hesitant HCWs (95%CI): 1.12 (1.08–1.16)** Black and Asian HCWs with higher COVID–19 CBS-scores: more vaccine hesitant vs. White HCWs**
Qunaibi et al. [50]	Worldwide	Cross-sectional	Arab-speaking HCWs	5708	30.6 (±10)	44.4%	NR	Vaccine acceptance rate: 26.7%. DC: January 2021	*“Coronavirus/vaccine is a conspiracy”: n* = *700 (12.3%)*
Usman et al. [51]	Pakistan	Cross-sectional	Undergraduate healthcare students	410	NR	46.8 %	NR	More than 70% of the participants wanted to be the first to get vaccinated DC: January-February 2021	*“Microchip implantation theory associated with Bill Gates” and “COVID–19 as a part of economic war between developed countries”: n=67 (16.4%)*
Elhadi et al. [52]	Libya	Cross-sectional	Physicians, medical students, paramedics	3967	30.6 (±9.8)	58.7%	NR	NR DC: December 2020	*“The novel corona virus is undoubtedly human-made to implement particular agendas”: n=1432 (36.1%)* *Medical Students: 34.9%* *Physicians: 34.1%* *Paramedic and nurses: 41.9%*
Szmyd et al. [46]	Poland	Cross-sectional	Medical students (dentistry, dietetics, emergency medical service, laboratory diagnostic, medicine, nursing, obstetric, pharmacy and physiotherapy student)	687	NR	64.77%	NR	91.99% planning to get vaccinated, 4.08% not planning to get vaccinated DC: December 2020	*“Belief in CTs (overall)”: n* = *59 (8.59%)* *“Belief in microchip injection”: n* = *12 (1.75%)* *“Belief in control of births by vaccine manufacturers”: n=5 (0.73%)*
Shehata et al. [53]	Egypt	Cross-sectional	Physicians working at various healthcare levels	1268	NR	59.4%	NR	36.7% did not accept to uptake the vaccine when available, 39% would wait for further review, while only 24.3% accept to uptake it. DC: March-May 2021	**“I think vaccination is a plot”: *n*** = **33 (2.6%)**
Al-Sanafi et al. [54]	Kuwait	Cross-sectional	Physicians, dentists, pharmacists, nurses, laboratory technicians, other (physiotherapists; dieticians and nutritionists; optometrists, etc.)	1019	34 (±9.7)	61.4%	Kuwait:75.1% Non-Kuwait: 21.7% Stateless/unknown: 3.2%	83.3% were vaccinated, 9% rejected vaccination, 7.7% answered maybe. DC: March 2021	*“COVID–19 has a human-made origin”: n* = *300 (29.4%)* Belief in “COVID–19 has a human made origin” (67.3%): more hesitancy vs. non-belief/no opinion** Higher VCBS score correlated with COVID–19 vaccine hesitancy** Rejection of vaccination (vs. hesitancy and acceptance) correlated with higher levels of CT** The dependence on social media platforms, TV programs, newspapers, and news releases correlated with higher VCBS (vs. scientists/scientific journals, doctors/other HCWs**
Castañeda-Vasquez et al. [55]	Mexico	Cross-sectional	Medical guild, nursing, dental, psychology, and laboratory personnel	543	NR	65%	NR	Participants were not yet vaccinated. Only 5.5% stated that they would reject the vaccine DC: October-December 2020	***“The vaccine is part of a worldwide conspiracy”: n=34 (6%)*** Higher CT beliefs (40%) among vaccine-hesitant HCWs; vs. belief in CTs among vaccine – acceptant HCWs*** [OR] Belief in CTs among vaccine – hesitant HCWs vs. belief in CTs and vaccine acceptance (95%CI): 14.879 (6.384–34.677)***
Kükrer et al. [56]	Turkey	Cross-sectional	Academic physicians, specialist physicians, family physicians, midwives, nurses, health technicians, health officers, and pharmacists in public and private institution hospitals	442	NR	66.5%	NR	10% will not get vaccinated. 20.1% vaccine hesitant DC: October – December 2020	*“I think it is the sheath theory of implanting traceable microchips in the bodies of millions of people with the vaccine microchip claimed in the media”: n= 14 (3.2%)*
Iliyasu et al. [57]	Nigeria	Mixed-method	Clinical staff (physician, nurse/midwife, pharmacist, laboratory scientist, physiotherapist; CHEO, ward attendant) and non-clinical staff (administrative, management, support service) at a tertiary referral hospital center	284	37.9 (± 10.36)	46.1%	Hausa/Fulani:82.04% Others:18.06%	NR DC: March 2021	*“Concerned about rumors of depopulation (or “population control’) and infertility related to COVID–19 vaccines”: n=150 (52.8%)* *HCWs believing in CTs but still willing to accept vaccination: 12.7%* *[OR] HCWs not believing in CTs (vs believing) and vaccine acceptance (95%CI): 2.55 (1.25–5.20)*

CBS, Conspiracy Belief Scale; CHEO, community health extension officers; CT, conspiracy theory; DC, timing of data collection; HCW, Healthcare Worker; (a)OR, (adjusted) Odds Ratio with coincidence interval of 95%; NR, not reported; NS, not significant; *p < 0.05, **p < 0.001, ***p < 0.0001; VCBS: Vaccine Conspiracy Belief Scale.

a : Dubov (2022) and Roberts (2022) extracted their data from Dubov (2021) for secondary analysis. Bold: prevalence of CTs regarding HCWs who are vaccine hesitant; Italic: prevalence of CTs regardless of vaccination status; bold and italic: combination of HCW CT believers who are vaccine hesitant and believe in CTs regardless of vaccination status.

### Prevalence and nature of COVID-19-related CTs among HCWs

Prevalence rates of COVID-19-related CTs among HCWs varied widely, ranging from 0.89 % [20] to 75.6 % [24] (average rate across 22 studies = 21.7%, median = 14.4). Although most of the included studies reported prevalence rates regardless of the vaccination status of HCWs, approximately one-third of these reported rates for vaccine-hesitant HCWs or rates separately for vaccinated and hesitant HCWs (see Table 1). The reported prevalence rates of COVID-19-related CTs mainly concern vaccine-hesitant HCWs (although certain studies have shown that a minority of vaccinated HCWs or HCWs who accepted getting vaccinated also endorse CTs) [27, 32, 55].

When comparing prevalence rates by geographical location, in general, higher rates of COVID-19-related CTs among HCWs were found in most countries of the Arab world. Studies conducted in Jordan, for example, consistently found 30% to 45.5% of their HCWs believed in CTs [26, 30, 37]. Studies performed in Sudan, Saudi Arabia, Kuwait, and Libya also found almost one-third to half of their HCWs believe in CTs [27, 38, 52, 54]. However, lower CT prevalence rates (2.6%-12.5%) were found in four other studies from the Arab World [25, 45, 50, 53]. Among African countries not belonging to the Arab world, the highest prevalence rates of CTs among HCWs were found in two studies from Ethiopia (30.1% and 75.6%) [24, 36] and one from Nigeria (52.8%) [57]. In the remaining African countries, less than 10 % of HCWs were found to believe in COVID-19-related CTs [21, 32, 47]. US studies showed heterogeneous results. While Dubov et al. found conspiracy prevalence rates up to 38 % among HCWs [44], no conspiracy thinking was found in the study by Hoffman et al. [34]. Prevalence rates of COVID-19-related CTs among European HCWs were less than 10% [35, 40, 46, 47, 49], except for one study conducted in Croatia and Bosnia where prevalence rates of CTs among medical students reached up to 46.4% [29].

While some of the included studies examined various specific COVID-19-related CTs, others did not differ between specific CTs. Although it therefore remains difficult to determine which types of CTs are more prevalent among HCWs in certain regions, compared to those in other regions, some patterns could be observed. While in European countries and Northern America, an increased belief of HCWs in the “destabilization and power gain” narrative was found [29, 35, 42–43, 46, 47], African HCWs particularly endorsed the “population reduction” and “liberty restriction” narratives [21, 22, 41, 48, 57] (see Table 2). The specific prevalence of various types of CTs along with detailed descriptions are found in Table 1.

**Table 2.** Types of COVID-19-related CTs (based on Fotakis & Simou, 2023) [69]

Types of COVID-19-related CTs	
Destabilization and power gain: *prevention and control measures were deployed as destabilizing actions for achieving financial or political power*	COVID–19 is a biological weapon from China to establish world order. Spread of the virus is a deliberate attempt by a group of powerful people to make money or to take control.
Population reduction: *the virus and vaccines were developed to reduce the global or specific population*	COVID–19 was intentionally created to reduce the world’s population or to get rid of certain groups of people. Vaccine is used to carry out mass sterilization.
Liberty restriction: *the virus and vaccines were developed to reduce liberty*	Vaccine contains microchips to control people. Vaccine is used to alter DNA structures. Coronavirus is just an excuse to suppress civil liberties.
Big pharma plot: *Big Pharma created the virus and/or is knowingly producing ineffective or harmful vaccine*	Big Pharma created coronavirus to profit from the vaccines. Vaccine’s effectiveness data are fabricated by Big Pharma.
5 G: *5 G networks promote the spread of COVID–19*	COVID–19 pandemic is induced by 5 G networks. 5 G cell phone technology is responsible for the spread of the coronavirus.
Non-existence: *COVID–19 does not exist*	Coronavirus is a hoax or a myth to force vaccinations on people.
Other	COVID–19 is a message from God. Bill Gates is behind the coronavirus pandemic.

### Determinants associated with CTs among HCWs

The majority of studies among HCWs did not investigate sociodemographic, psychological, religious, or political determinants of CTs. Moreover, heterogeneous results were found.

#### Sociodemographic determinants

Only three studies investigated the relationship between gender and CTs [21, 39, 40]. Of these, Petersen et al. found that women tended more towards CTs than men (*p*<0.001) [40]. Although Oyeyemi et al. found men to be statistically more likely to believe in “DNA alteration theory” than women, results between genders were not significant for the “microchip injection theory” [21]. Jamil et al. found no correlation between these variables [39].

Two studies investigating the relationship between age and CTs did not find an age-related effect [12, 40].

Regarding race and ethnicity, the study of Odejinmi et al. found no significant association between ethnicity and conspiracy thinking [35]. Woolf et al. however, found Black and Asian HCWs having higher scores on the COVID-19 conspiracy beliefs scale than White people (*p* < 0.001) [49]. Moreover, in the US study of Dubov et al., CTs were more widespread among Hispanic HCWs than among Asian-American and African-American HCWs. These groups, however, were not compared with White HCWs [43].

Several studies found an association between educational level or profession and conspiracy endorsement. Kaya et al. demonstrated that HCWs with higher educational levels (master’s and doctorate degrees) believed significantly less in CTs, in comparison to HCWs with a bachelor degree and lower educational level [12]. In general, it seems that particularly nurses and non-clinical and administrative staff stand out as having significantly higher levels of CT beliefs. For example, in a German study, CTs were found to be significantly more prevalent among nursing, medical technical, and administrative staff, in comparison to physicians and scientific staff [40]. In a study from Nigeria, nurses were significantly more likely to believe in CTs than physicians [22].

#### Political orientation, government trust, information sources, and religious beliefs

A U.S. study found that the group of HCWs who had the highest rate of CTs were lean Republicans while the group with the lowest CTs rates were Democrats [44]. One study conducted in Nigeria showed that the odds of believing in the microchip theory increased significantly with a decreasing level of trust in the government’s information regarding the COVID-19 pandemic and vaccines (odds ratio [OR] 4.6, 95% CI 2.6–8.0), when compared to those with a high level of trust. Findings were similar for those who believed in the DNA alteration theory (OR 5.2, 95% CI 3.1–8.8) [22].

Regarding information sources, HCWs who were more dependent on social media, TV programs, and popular newspapers had a higher score on the Vaccine Conspiracy Belief Scale, compared to those who relied on information provided by scientists, doctors (or HCWs in general), or scientific journals [54]. In line with these findings, Oyeyemi et al. found HCWs using health authorities as the main source of information to be less likely to believe in CTs about microchips (OR 0.4, 95% CI 0.2–0.7) and the “DNA alteration theory” (OR 0.5, 95% CI 0.3–0.9) [22].

No study was found examining the relationship between religion and CTs among HCWs.

#### Psychological aspects

One large international study (*n* = 12,792) suggested that HCWs with current depressive symptoms had a higher overall tendency to believe in CTs [28].

## Discussion

Our scoping review has shown that HCWs are not immune to CTs. Although prevalence rates of COVID-19-related CTs varied considerably (ranging from 0.89% to 75.6%), they generally appeared to be higher among HCWs in most countries of the Arab world, Ethiopia, and Nigeria, in comparison to those in other African and most Western countries. Limited and heterogeneous data prevented conclusive findings on determinants associated with CTs among HCWs. The only consistent observation was that HCWs with higher educational attainment tend to endorse CTs less frequently.

The wide variance in prevalence rates of COVID-19-related CTs among HCWs is in line with the results that have been found in the general population (prevalence rates ranging from 0.4% to 82.7%) [58, 59]. Despite this wide range, our results suggest that geographical variations exist, with higher prevalence rates in most countries of the Arab world and some countries on the African continent. One potential explanation for this phenomenon is the instability in most of these regions, stemming from political, economic, and/or religious conflicts, as well as natural disasters [22, 39, 60–62]. To date, studies have identified two nation-level variables that consistently predict CTs across multiple datasets: lack of economic vitality and the presence of corrupted undemocratic regimes. Thus people will believe CTs more when their perceptions of current and future economic performance within their nation are relatively poor, and when electoral processes are distorted, civil liberties restricted, and official media are mouthpieces for government propaganda [63] This results in ineffective governance and initiatives, fostering mistrust and leading to a conspiracy mentality. Another potential explanation is that nations that are high in collectivism are also more likely to endorse CTs. Collectivist cultures (and collectivism-oriented individuals) are more likely to make relational explanations when attributing causality to ambiguous events, which in turn could lead to CT endorsement [63]. Finally, historical (or even present) marginalization of certain groups of people or historical examples of abuse (e.g., unethical practices by pharmaceutical companies) may make CTs attractive in these countries [22, 64–66]. In European countries, the prevalence of COVID-19-related CTs among HCWs remained under 10% [35, 40, 46, 47, 49], which is in line with the results that have been reported by the ECDC (European Centre for Disease Prevention and Control) [67]. Western countries usually are economically and politically more stable. However, the recent shift towards more radical right-wing political authoritarian orientations could become a fueling factor for endorsing more CTs [63, 68]. Certain patterns in the prevalence of specific types of CTs among HCWs were observed in particular regions, aligning with the findings of Fotakis’s study on the general population. For example, medical students in Bosnia and Croatia exhibited a strong belief in “Big Pharma plots” [29], a trend also noted in the general population across the Balkan region [69].

As mentioned above, limited and heterogeneous data prevented conclusive findings on determinants associated with CTs among HCWs. Studies investigating age and gender-related associations with conspiracy thinking in HCWs generally found no significant relationship. A recent large-scale study, including data from 21 different countries, only found age to be (negatively) correlated with conspiracy thinking [64]. Although our data on race and ethnicity are difficult to interpret, in general, it is known that CTs flourish particularly among cohesive minority groups that are suppressed by a dominant majority coalition [58, 70]. The above-mentioned large-scale, multicultural study found Black identification to be positively related to conspiracy thinking [64]. Regarding the level of education, three studies were found showing that HCWs with higher educational levels (master’s and doctorate degrees) believed significantly less in CTs, in comparison to HCWs with bachelor’s degrees and lower educational levels (nurses, medical-technical and administrative staff) [12, 22, 40]. These results are in line with the results of studies on vaccine hesitancy that have been conducted in HCWs [7]. Particularly the finding regarding nurses raises concerns as these are involved in many different aspects of immunization and often provide direct care to patients with COVID-19.

Only one study included in our review examined the relationship between psychological factors and CTs among HCWs, finding that HCWs with current depressive symptoms have higher CT rates [28]. Studies among the general population, however, have also shown that personality traits such as low tolerance for uncertainty and ambiguity, impulsivity, low perceived risk, lower analytical thinking, and negative emotions are significantly associated with belief in CTs [58, 71, 72]. Several studies have found that people who score higher on CT belief scales also score higher on self-report measures of schizotypal personality traits and paranoid ideation. An important side note is that CTs are not reducible to paranoia; the main difference is that CTs focus mostly on elite groups and are convinced they attack a specific population, whereas paranoid people tend to see themselves as a target [63].

Our study shows that most HCWs who believe in CTs, are also vaccine hesitant. As in general, studies consistently report a significant negative association between belief in COVID-19-related CTs and vaccination intention or uptake [73].

Vaccination hesitancy among HCWs not only poses a threat to global health efforts fighting the COVID-19 pandemic, it may also fuel public fear and erode trust in the healthcare system [42, 74]. Therefore, the following recommendations can be implemented to reduce the likelihood of CTs among HCWs.

Delivering counterarguments to people before they encounter CTs (i.c. prebunking), has been shown to increase vaccine willingness, compared to people already exposed to CTs [75, 76, 77]. Moreover, exposing the manipulative persuasion tactics used to spread CTs (such as the use of emotional language, misleading rhetoric, or fake experts that sow doubt about the scientific consensus) may also reduce the likelihood of adapting CTs [75, 77]. Another effective preventive approach is to encourage people to be more critical consumers of CTs before they are first exposed to these by stimulating metacognitive reflection or critical thinking [75, 77, 78].

Once they are established, health-related CTs may be extremely resistant to correction [79]. Confrontation by simply presenting fact-based anti-conspiracy arguments may even strengthen CTs [80, 81]. Although an open-minded approach through the use of empathy and active listening by inviting the person towards a deeper examination of the building bricks of their CTs is more productive [79, 80], it only showed small effects [82, 83]. Thus, simply giving people the “right” set of facts does not guarantee that they will adopt desirable beliefs or engage in advisable behaviors. One must also recognize the role of people’s motivations in believing these theories [64]. Many people with CTs incorrectly believe that their hesitancy to be vaccinated is rather common and overestimate how much others believe anti-vaccine CTs. One therefore should highlight that CTs are not as commonplace as they may think, for example by using normative feedback^1^, preferably in the context of a relevant social group [76, 79, 84]. Healthcare leaders could act as role models by being a trusted source of information and creating new social norms by getting publicly vaccinated and explicitly expressing the benefits of vaccination. This way, they can convey through their actions that getting vaccinated is safe and beneficial and connect it to a shared collective identity and enhance feelings of control and self-efficacy of their employees [76].

Several authors endorse the use of vaccine mandates to lessen the deleterious effects of CTs [76, 85]. Although mandatory vaccination interferes with the right to private life, the exceptions under Article 8 of the European Convention on Human Rights (in particular the protection of public health and the protection of the rights and freedom of others) might justify these interferences [86]. Moreover, fear of social sanctions can be a powerful motivator. Although this approach has been shown effective [87], it does not target vaccine hesitancy and may actually arouse suspicions, thereby encouraging CTs [64].

Regardless of the above-mentioned recommendations, it is important to know that HCWs holding CTs probably are not a homogeneous group. Research has shown that next to COVID-19 conspiracy “believers” and “non-believers”, there also exist COVID-19 conspiracy “ambivalent believers” (i.c. vaccine hesitant COVID-19 CT believers who are less likely to believe CTs than COVID-19 conspiracy “believers” as they are less misinformed or uninformed about the COVID-19 vaccine. This explains why this group is more uncertain, ambivalent, and undecided about the COVID-19 vaccine than the “believers”). All these groups differ in terms of psychological characteristics [42, 44, 88]. The need to tailor interventions for HCWs believing in COVID-19 CTs therefore is necessary.

### Strengths and limitations

A key strength of this analysis is the extensive search strategies including several databases (see Supplementary Material). One major limitation of this study is the exclusion of qualitative data, which give the opportunity to understand more deeply why HCWs believe in CTs. Moreover, heterogeneity across studies in terms of tools, methods, and survey designs made it hard to perform a thorough quantitative analysis of the data. Although we didn’t critically appraise the included studies, we also noticed that several of these studies were poorly performed. Furthermore, we surmise that the actual number of HCWs with conspiracy beliefs may be higher than our results indicate. There may be unidentified “unspoken vaccine hesitancy” cases, a phenomenon where HCWs do not express publicly their hesitancy and potentially conspiratorial concerns about vaccines due to institutional and societal pressure and out of fear of being mocked or stigmatized [89]. Finally, the majority of the included studies had a cross-sectional design, which does not allow us to infer causal relationships.

## Conclusion

Although COVID-19-related CTs may be highly prevalent among HCWs, gaps in understanding the drivers of CTs among HCWs remain. Given HCWs’ critical role in public health, especially during pandemics, further research is therefore essential to mitigate the impact of CTs on vaccine willingness among HCWs.

## Supporting information

Loyens et al. supplementary material Loyens et al. supplementary material

## Data Availability

The analysis is based on the content of the selected publications.

## Footnotes

1 intervention designed to correct misperceptions regarding the prevalence of problematic behavior by showing individuals engaging in such behaviors that their own behavior is atypical with respect to actual norms.

## Supplementary material

The supplementary material for this article can be found at http://doi.org/10.1192/j.eurpsy.2025.12.
