# Supplementary material for: Prevalence, nature, and determinants of COVID-19-related conspiracy theories among healthcare workers: a scoping review
Source: Eur Psychiatry. 2025 Mar 3;68(1):e62. doi: 10.1192/j.eurpsy.2025.12 (PMC12188351; doi:10.1192/j.eurpsy.2025.12)
Supplement: Loyens et al. supplementary material [file S0924933825000124sup001.pdf]

Supplementary materials

Search string

TOTAAL = 12538 HITS

PUBMED (2671 hits)

EMBASE (3983 hits)

WEB OF SCIENCE (2749 hits)

CINAHL (502 hits)

SCOPUS (2633 hits)

DUPLICATES (7539 articles)

SCREENING (5018 articles)

PUBMED (2671 hits, 03/10/2023)

(("Coronavirus"[Mesh:NoExp] OR "Betacoronavirus"[Mesh:NoExp] OR "Coronavirus Infections"[Mesh:NoExp] OR "COVID-19"[MeSH] OR nCoV[tiab] OR 2019nCoV[tiab] OR COVID[tiab] OR COVID19[tiab] OR nCoV[ad] OR 2019nCoV[ad] OR COVID[ad] OR COVID19[ad] OR "SARS-Cov-2"[MeSH] OR SARS2[ad] OR "cov 2"[ad] OR cov2[ad] OR coronavirus*[ad] OR "corona virus*"[ad] OR SARS2[tiab] OR "cov 2"[tiab] OR cov2[tiab] OR coronavirus*[tiab] OR "corona virus*"[tiab] OR "Wuhan virus"[tiab] OR ((wuhan[tiab] OR novel[tiab] OR new[tiab] OR 19[tiab] OR 2019[tiab] OR epidem*[tiab] OR pandem*[tiab] OR outbreak[tiab] OR wuhan[ad] OR novel[ad] OR new[ad] OR 19[ad] OR 2019[ad] OR epidemy[ad] OR epidemic*[ad] OR pandem*[ad] OR outbreak[ad]) AND ("pneumonia virus*"[tiab] OR cov[tiab] OR hcov[tiab]))) AND ("COVID-19 Vaccines"[Mesh] OR "Vaccination"[Mesh] OR "Vaccination Refusal"[Mesh] OR "vaccin*"[tiab])) AND ("conspira*"[tiab] OR "belie*"[tiab] OR "health-related belief*"[tiab] OR "Anti-Vaccination Movement"[Mesh] OR "antivaccin*"[tiab] OR "anti-vaccin*"[tiab] OR "anti-vaxxer*"[tiab] OR "CTs*"[tiab] OR "denial*"[tiab] OR "refusal*"[tiab] OR "defiance"[tiab] OR "mistrust"[tiab] OR "rumor*"[tiab] OR "plot*"[tiab] OR "hesitan*"[tiab] OR "accept*"[tiab] OR "reject*"[tiab] OR "willingness"[tiab] OR "intent*"[tiab] OR "antiscience*"[tiab] OR "fake science*"[tiab] OR "science denial*"[tiab] OR "science literac*"[tiab] OR "scientific illiterac*"[tiab] OR "scientifically illiterate"[tiab] OR "misinformation"[tiab] OR "disinformation"[tiab] OR "misleading"[tiab] OR "misinterpretation*"[tiab] OR "misconception*"[tiab] OR "worldview*"[tiab] OR "fake news*"[tiab] OR "myth*"[tiab] OR "prejudice*"[tiab] OR "false news*"[tiab] OR "fake information"[tiab] OR "information overload"[tiab] OR "infodemic*"[tiab] OR "misinfodemic*"[tiab] OR "infodemiolog*"[tiab] OR "debunk*"[tiab] OR "hoax"[tiab] OR "5G"[tiab] OR "Bill Gates"[tiab] OR "ozone"[tiab] OR "bioweapon*"[tiab] OR "bio-weapon*"[tiab] OR "lobby"[tiab] OR "right-wing"[tiab] OR "xenophobi*"[tiab] OR "paranoia*"[tiab]) AND ("Health Personnel"[Mesh] OR "health personnel"[tiab] OR "health

care personnel"[tiab] OR "healthcare personnel"[tiab] OR "health care assistant*"[tiab] OR "healthcare assistant*"[tiab] OR "health profession personnel"[tiab] OR "health professional*"[tiab] OR "health and hygiene professional*"[tiab] OR "practice professional*"[tiab] OR "medicine professional*"[tiab] OR "health care provider*"[tiab] OR "healthcare provider*"[tiab] OR "healthcare worker*"[tiab] OR "health care worker*"[tiab] OR "health worker*"[tiab] OR "healthcare professional*"[tiab] OR "health care professional*"[tiab] OR "health care practitioner*"[tiab] OR "healthcare practitioner*"[tiab] OR "paramedical practitioner*"[tiab] OR "physiotherapist*"[tiab] OR "clinician*"[tiab] OR "clinical practitioner*"[tiab] OR "home health aide*"[tiab] OR "public health officer*"[tiab] OR "nurse*"[tiab] OR "nursing*"[tiab] OR "midwife*"[tiab] OR "midwive*"[tiab] OR "mental health personnel"[tiab] OR "medical personnel"[tiab] OR "medical staff"[tiab] OR "medical doctor*"[tiab] OR "medical technician*"[tiab] OR "medical assistant*"[tiab] OR "hospital personnel"[tiab] OR "laboratory personnel"[tiab] OR "health educator*"[tiab] OR "pharmacist*"[tiab] OR "physician*"[tiab] OR "dentist*"[tiab] OR "dental*"[tiab] OR "dietitian*"[tiab] OR "dietician*"[tiab] OR "nutritionist*"[tiab] OR "dietetic*"[tiab] OR "librarian*"[tiab] OR "library*"[tiab] OR "audiolog*"[tiab] OR "speech therapist*"[tiab] OR "optometrist*"[tiab] OR "optician*"[tiab] OR "laboratory work*"[tiab] OR "ambulance work*"[tiab] OR "preventionist*"[tiab] OR "occupational therap*"[tiab] OR "physical therap*"[tiab] OR "physician assistant*"[tiab] OR "recreational therapist*"[tiab] OR "social work*"[tiab] OR "surgeon*"[tiab] OR "surgical*"[tiab] OR "case manager*"[tiab] OR "chiropractor*"[tiab] OR "chiropractic*"[tiab] OR "coroner*"[tiab] OR "pharmacist*"[tiab] OR "information technology personnel"[tiab] OR "computer scientist*"[tiab] OR "IT specialist*"[tiab] OR "psychiatrist*"[tiab] OR "psychologist*"[tiab] OR "psychotherapist*"[tiab] OR "midwi*"[tiab] OR "osteopath*"[tiab] OR "personal trainer*"[tiab] OR "personal care worker*"[tiab] OR "health service manager*"[tiab] OR "health service provider*"[tiab] OR "life science professional*"[tiab] OR "biomedical engineer*"[tiab] OR "medical secretar*"[tiab] OR "Students, Health Occupations"[Mesh] OR "health occupation student*"[tiab] OR "health occupations student*"[tiab] OR "medical student*"[tiab] OR "medical school student*"[tiab] OR "med school student*"[tiab] OR "med student*"[tiab] OR "premedical student*"[tiab] OR "pre-medical student*"[tiab] OR "paramedical student*"[tiab] OR "para-medical student*"[tiab] OR "healthcare student*"[tiab] OR "health care student*"[tiab] OR "pupil nurse*"[tiab] OR "physical therapy student*"[tiab] OR "respiratory therapy student*"[tiab] OR "midwifery student*"[tiab] OR "midwifery trainee*"[tiab] OR "chiropractic student*"[tiab] OR "allied health student*"[tiab] OR "pharmacy student*"[tiab] OR "student pharmacist*"[tiab] OR "pharmaceutical student*"[tiab] OR "physiotherapy student*"[tiab] OR "health student*"[tiab])

EMBASE (3983 hits, 03/10/2023)

(('Coronavirinae'/de OR 'Betacoronavirus'/de OR 'Coronavirus infection'/de OR 'coronavirus disease 2019'/exp OR 'nCoV':ti,ab,kw,ff OR '2019nCoV':ti,ab,kw,ff OR 'COVID':ti,ab,kw,ff OR 'COVID19':ti,ab,kw,ff OR 'Severe acute respiratory syndrome coronavirus 2'/exp OR 'severe acute respiratory syndrome 2':ti,ab,kw,ff OR 'SARS2':ti,ab,kw,ff OR 'cov 2':ti,ab,kw,ff OR 'cov2':ti,ab,kw,ff OR 'coronavirus*':ti,ab,kw,ff OR 'betacoronavirus*':ti,ab,kw,ff OR 'coronavirinae':ti,ab,kw OR 'corona virus*':ti,ab,kw,ff OR 'Wuhan virus':ti,ab,kw OR (('wuhan':ti,ab,kw,ff OR 'novel':ti,ab,kw,ff OR 'new':ti,ab,kw,ff OR '19':ti,ab,kw,ff OR

'2019':ti,ab,kw,ff OR 'epidem*':ti,ab,kw OR 'pandem*':ti,ab,kw,ff OR 'outbreak':ti,ab,kw,ff OR 'epidemy':ff OR 'epidemic*':ff) AND ('pneumonia virus*':ti,ab,kw OR 'cov':ti,ab,kw OR 'hcov':ti,ab,kw))) AND ('SARS-CoV-2 vaccine'/exp OR 'vaccination'/exp OR 'vaccination refusal'/exp OR 'vaccine hesitancy'/exp OR 'vaccin*':ti,ab,kw)) AND ('conspiracy theory'/exp OR 'conspira*':ti,ab,kw OR 'belie*':ti,ab,kw OR 'health-related belief*':ti,ab,kw OR 'anti-vaccination movement'/exp OR 'antivaccin*':ti,ab,kw OR 'anti-vaccin*':ti,ab,kw OR 'anti-vaxxer*':ti,ab,kw OR 'CTs*':ti,ab,kw OR 'denial*':ti,ab,kw OR 'refusal*':ti,ab,kw OR 'defiance':ti,ab,kw OR 'mistrust':ti,ab,kw OR 'rumor*':ti,ab,kw OR 'plot*':ti,ab,kw OR 'hesitan*':ti,ab,kw OR 'accept*':ti,ab,kw OR 'reject*':ti,ab,kw OR 'willingness':ti,ab,kw OR 'intent*':ti,ab,kw OR 'antiscience*':ti,ab,kw OR 'fake science*':ti,ab,kw OR 'science denial*':ti,ab,kw OR 'science literac*':ti,ab,kw OR 'scientific illiterac*':ti,ab,kw OR 'scientifically illiterate':ti,ab,kw OR 'misinformation':ti,ab,kw OR 'disinformation':ti,ab,kw OR 'misleading':ti,ab,kw OR 'misinterpretation*':ti,ab,kw OR 'misconception':ti,ab,kw OR 'worldview*':ti,ab,kw OR 'fake news*':ti,ab,kw OR 'false news*':ti,ab,kw OR 'myth*':ti,ab,kw OR 'prejudice*':ti,ab,kw OR 'fake information':ti,ab,kw OR 'information overload':ti,ab,kw OR 'infodemic*':ti,ab,kw OR 'misinfodemic*':ti,ab,kw OR 'infodemiolog*':ti,ab,kw OR 'debunk*':ti,ab,kw OR 'hoax':ti,ab,kw OR '5G':ti,ab,kw OR 'Bill Gates':ti,ab,kw OR 'ozone':ti,ab,kw OR 'bioweapon*':ti,ab,kw OR 'bio-weapon*':ti,ab,kw OR 'lobby':ti,ab,kw OR 'right-wing':ti,ab,kw OR 'xenophobi*':ti,ab,kw OR 'paranoia*':ti,ab,kw) AND ('health care personnel'/exp OR 'health personnel':ti,ab,kw OR 'health care personnel':ti,ab,kw OR 'healthcare personnel':ti,ab,kw OR 'health care assistant*':ti,ab,kw OR 'healthcare assistant*':ti,ab,kw OR 'health profession personnel':ti,ab,kw OR 'health professional*':ti,ab,kw OR 'health and hygiene professional*':ti,ab,kw OR 'practice professional*':ti,ab,kw OR 'medicine professional*':ti,ab,kw OR 'health care provider*':ti,ab,kw OR 'healthcare provider*':ti,ab,kw OR 'healthcare worker*':ti,ab,kw OR 'health care worker*':ti,ab,kw OR 'health worker*':ti,ab,kw OR 'healthcare professional*':ti,ab,kw OR 'health care professional*':ti,ab,kw OR 'health care practitioner*':ti,ab,kw OR 'healthcare practitioner*':ti,ab,kw OR 'paramedical practitioner*':ti,ab,kw OR 'physiotherapist*':ti,ab,kw OR 'clinician*':ti,ab,kw OR 'clinical practitioner*':ti,ab,kw OR 'home health aide*':ti,ab,kw OR 'public health officer*':ti,ab,kw OR 'nurse*':ti,ab,kw OR 'nursing*':ti,ab,kw OR 'midwife*':ti,ab,kw OR 'midwive*':ti,ab,kw OR 'mental health personnel':ti,ab,kw OR 'medical personnel':ti,ab,kw OR 'medical staff':ti,ab,kw OR 'medical doctor*':ti,ab,kw OR 'medical technician*':ti,ab,kw OR 'medical assistant*':ti,ab,kw OR 'hospital personnel':ti,ab,kw OR 'laboratory personnel':ti,ab,kw OR 'health educator*':ti,ab,kw OR 'pharmacist*':ti,ab,kw OR 'physician*':ti,ab,kw OR 'dentist*':ti,ab,kw OR 'dental*':ti,ab,kw OR 'dietitian*':ti,ab,kw OR 'dietician*':ti,ab,kw OR 'nutritionist*':ti,ab,kw OR 'dietetic*':ti,ab,kw OR 'librarian*':ti,ab,kw OR 'library*':ti,ab,kw OR 'audiolog*':ti,ab,kw OR 'speech therapist*':ti,ab,kw OR 'optometrist*':ti,ab,kw OR 'optician*':ti,ab,kw OR 'laboratory work*':ti,ab,kw OR 'ambulance work*':ti,ab,kw OR 'preventionist*':ti,ab,kw OR 'occupational therap*':ti,ab,kw OR 'physical therap*':ti,ab,kw OR 'physician assistant*':ti,ab,kw OR 'recreational therapist*':ti,ab,kw OR 'social work*':ti,ab,kw OR 'surgeon*':ti,ab,kw OR 'surgical*':ti,ab,kw OR 'case manager*':ti,ab,kw OR 'chiropractor*':ti,ab,kw OR 'chiropractic*':ti,ab,kw OR 'coroner*':ti,ab,kw OR 'pharmacist*':ti,ab,kw OR 'information technology personnel':ti,ab,kw OR 'computer scientist*':ti,ab,kw OR 'IT specialist*':ti,ab,kw OR 'psychiatrist*':ti,ab,kw OR 'psychologist*':ti,ab,kw OR 'psychotherapist*':ti,ab,kw OR 'midwive*':ti,ab,kw OR 'osteopath*':ti,ab,kw OR 'personal trainer*':ti,ab,kw OR 'personal care worker*':ti,ab,kw OR

'health service manager*':ti,ab,kw OR 'health service provider*':ti,ab,kw OR 'life science professional*':ti,ab,kw OR 'biomedical engineer*':ti,ab,kw OR 'medical secretar*':ti,ab,kw OR 'health student'/exp OR 'health occupation student*':ti,ab,kw OR 'health occupations student*':ti,ab,kw OR 'medical student*':ti,ab,kw OR 'medical school student*':ti,ab,kw OR 'med school student*':ti,ab,kw OR 'med student*':ti,ab,kw OR 'premedical student*':ti,ab,kw OR 'pre-medical student*':ti,ab,kw OR 'paramedical student*':ti,ab,kw OR 'para-medical student*':ti,ab,kw OR 'healthcare student*':ti,ab,kw OR 'health care student*':ti,ab,kw OR 'pupil nurse*':ti,ab,kw OR 'respiratory therapy student*':ti,ab,kw OR 'midwifery student*':ti,ab,kw OR 'midwifery trainee*':ti,ab,kw OR 'chiropractic student*':ti,ab,kw OR 'allied health student*':ti,ab,kw OR 'pharmacy student*':ti,ab,kw OR 'student pharmacist*':ti,ab,kw OR 'pharmaceutical student*':ti,ab,kw OR 'physiotherapy student*':ti,ab,kw OR 'physical therapy student*':ti,ab,kw OR 'health student*':ti,ab,kw)

WEB OF SCIENCE (2749 hits, 03/10/2023)

(TS=(("coronavirus*" OR "betacoronavirus*" OR "COVID-19" OR "coronavirinae" OR "nCoV" OR "2019nCoV" OR "COVID" OR "COVID19" OR "severe acute respiratory syndrome 2" OR "SARS2" OR "cov 2" OR "cov2" OR "corona virus*" OR "Wuhan virus" OR (("wuhan" OR "novel" OR "new" OR "19" OR "2019" OR epidem* OR pandem* OR "outbreak") AND ("pneumonia virus*" OR "cov" OR "hcov"))) OR AD=("nCoV" OR "2019nCoV" OR "COVID" OR "COVID19" OR "SARS2" OR "cov 2" OR "cov2" OR "coronavirus*" OR "betacoronavirus*" OR "corona virus*" OR "severe acute respiratory syndrome 2") OR (AD=("wuhan" OR "novel" OR "new" OR "19" OR "2019" OR "epidemy" OR "epidemic*" OR "pandem*" OR "outbreak"))) AND TS=("vaccin*") AND TS=("conspira*" OR "belie*" OR "health-related belief*" OR "antivaccin*" OR "anti-vaccin*" OR "anti-vaxxer*" OR "CTs*" OR "denial*" OR "refusal*" OR "defiance" OR "mistrust" OR "rumor*" OR "plot*" OR "hesitan*" OR "accept*" OR "reject*" OR "willingness" OR "intent*" OR "antiscience*" OR "fake science*" OR "science denial*" OR "science literac*" OR "scientific illiterac*" OR "scientifically illiterate" OR "misinformation" OR "disinformation" OR "misleading" OR "misinterpretation*" OR "misconception*" OR "worldview*" OR "fake news*" OR "myth*" OR "prejudice*" OR "false news*" OR "fake information" OR "information overload" OR "infodemic*" OR "misinfodemic*" OR "infodemiolog*" OR "debunk*" OR "hoax" OR "5G" OR "Bill Gates" OR "ozone" OR "bioweapon*" OR "bio-weapon*" OR "lobby" OR "right-wing" OR "xenophobi*" OR "paranoia*") AND TS=("health personnel" OR "health care personnel" OR "healthcare personnel" OR "health care assistant*" OR "healthcare assistant*" OR "health profession personnel" OR "health professional*" OR "health and hygiene professional*" OR "practice professional*" OR "medicine professional*" OR "health care provider*" OR "healthcare provider*" OR "healthcare worker*" OR "health care worker*" OR "health worker*" OR "healthcare professional*" OR "health care professional*" OR "health care practitioner*" OR "healthcare practitioner*" OR "paramedical practitioner*" OR "physiotherapist*" OR "clinician*" OR "clinical practitioner*" OR "home health aide*" OR "public health officer*" OR "nurse*" OR "nursing*" OR "midwife*" OR "midwive*" OR "mental health personnel" OR "medical personnel" OR "medical staff" OR "medical doctor*" OR "medical technician*" OR "medical assistant*" OR "hospital personnel" OR "laboratory personnel" OR "health educator*" OR "pharmacist*" OR "physician*" OR "dentist*" OR "dental*" OR "dietitian*"

OR "dietician*" OR "nutritionist*" OR "dietetic*" OR "librarian*" OR "library*" OR "audiolog*" OR "speech therapist*" OR "optometrist*" OR "optician*" OR "laboratory work*" OR "ambulance work*" OR "preventionist*" OR "occupational therap*" OR "physical therap*" OR "physician assistant*" OR "recreational therapist*" OR "social work*" OR "surgeon*" OR "surgical*" OR "case manager*" OR "chiropractor*" OR "chiropractic*" OR "coroner*" OR "pharmacist*" OR "information technology personnel" OR "computer scientist*" OR "IT specialist*" OR "psychiatrist*" OR "psychologist*" OR "psychotherapist*" OR "midwive*" OR "osteopath*" OR "personal trainer*" OR "personal care worker*" OR "health service manager*" OR "health service provider*" OR "life science professional*" OR "biomedical engineer*" OR "medical secretar*" OR "health occupation student*" OR "health occupations student*" OR "medical student*" OR "medical school student*" OR "med school student*" OR "med student*" OR "premedical student*" OR "pre-medical student*" OR "paramedical student*" OR "para-medical student*" OR "healthcare student*" OR "health care student*" OR "pupil nurse*" OR "physical therapy student*" OR "respiratory therapy student*" OR "midwifery student*" OR "midwifery trainee*" OR "chiropractic student*" OR "allied health student*" OR "pharmacy student*" OR "student pharmacist*" OR "pharmaceutical student*" OR "physiotherapy student*" OR "health student*")

CINAHL (502 hits, 03/10/2023)

((MH "Coronavirus") OR (MH "Coronavirus Infections") OR (MH "COVID-19") OR (MH "SARS-CoV-2") OR (MH "COVID-19 Pandemic") OR TI ("coronavirinae" OR "nCoV" OR "2019nCov" OR "COVID" OR "COVID19" OR "severe acute respiratory syndrome 2" OR "SARS2" OR "cov 2" OR "cov2" OR "coronavirus*" OR "betacoronavirus*" OR "corona virus*" OR "Wuhan virus") OR AB ("coronavirinae" OR "nCoV" OR "2019nCov" OR "COVID" OR "COVID19" OR "severe acute respiratory syndrome 2" OR "SARS2" OR "cov 2" OR "cov2" OR "coronavirus*" OR "betacoronavirus*" OR "corona virus*" OR "Wuhan virus") OR AF ("nCoV" OR "2019nCov" OR "COVID" OR "COVID19" OR "severe acute respiratory syndrome 2" OR "SARS2" OR "cov 2" OR "cov2" OR "coronavirus*" OR "betacoronavirus*" OR "corona virus*") OR ((TI ("wuhan" OR "novel" OR "new" OR "19" OR "2019" OR "epidem*" OR "pandem*" OR "outbreak") OR AB ("wuhan" OR "novel" OR "new" OR "19" OR "2019" OR "epidem*" OR "pandem*" OR "outbreak") OR AF ("wuhan" OR "novel" OR "new" OR "19" OR "2019" OR "epidemy" OR "epidemic*" OR "pandem*" OR "outbreak")) AND (TI ("pneumonia virus*" OR "cov" OR "hcov") OR AB ("pneumonia virus*" OR "cov" OR "hcov")))) AND ((MM "COVID-19 Vaccines") OR (MH "Immunization+") OR (MH "Vaccination Refusal+") OR "vaccin*") AND ((MM "Anti-Vaccination Movement") OR TI ("conspira*" OR "belie*" OR "health-related belief*" OR "antivaccin*" OR "anti-vaccin*" OR "anti-vaxxer*" OR "CTs*" OR "denial*" OR "refusal*" OR "defiance" OR "mistrust" OR "rumor*" OR "plot*" OR "hesitan*" OR "accept*" OR "reject*" OR "willingness" OR "intent*" OR "antiscience*" OR "fake science*" OR "science denial*" OR "science literac*" OR "scientific illiterac*" OR "scientifically illiterate" OR "misinformation" OR "disinformation" OR "misleading" OR "misinterpretation*" OR "misconception" OR "worldview*" OR "fake news*" OR "false news*" OR "fake

information" OR "information overload" OR "infodemic*" OR "misinfodemic*" OR "infodemiolog*" OR "debunk*" OR "hoax" OR "5G" OR "Bill Gates" OR "ozone" OR "bioweapon*" OR "bio-weapon*" OR "lobby" OR "right-wing" OR "xenophobi*" OR "paranoia*") OR AB ("conspira*" OR "belie*" OR "health-related belief*" OR "antivaccin*" OR "anti-vaccin*" OR "anti-vaxxer*" OR "CTs*" OR "denial*" OR "refusal*" OR "defiance" OR "mistrust" OR "rumor*" OR "plot*" OR "hesitan*" OR "accept*" OR "reject*" OR "willingness" OR "intent*" OR "antiscience*" OR "fake science*" OR "science denial*" OR "science literac*" OR "scientific illiterac*" OR "scientifically illiterate" OR "misinformation" OR "disinformation" OR "misleading" OR "misinterpretation*" OR "misconception" OR "worldview*" OR "fake news*" OR "false news*" OR "myth*" OR "prejudice*" OR "fake information" OR "information overload" OR "infodemic*" OR "misinfodemic*" OR "infodemiolog*" OR "debunk*" OR "hoax" OR "5G" OR "Bill Gates" OR "ozone" OR "bioweapon*" OR "bio-weapon*" OR "lobby" OR "right-wing" OR "xenophobi*" OR "paranoia*")) AND ((MH "Health Personnel+") OR (MH "Students, Health Occupations+") OR TI ("health personnel" OR "health care personnel" OR "healthcare personnel" OR "health care assistant*" OR "healthcare assistant*" OR "health profession personnel" OR "health professional*" OR "health and hygiene professional*" OR "practice professional*" OR "medicine professional*" OR "health care provider*" OR "healthcare provider*" OR "healthcare worker*" OR "health care worker*" OR "health worker*" OR "healthcare professional*" OR "health care professional*" OR "health care practitioner*" OR "healthcare practitioner*" OR "paramedical practitioner*" OR "physiotherapist*" OR "clinician*" OR "clinical practitioner*" OR "home health aide*" OR "public health officer*" OR "nurse*" OR "nursing*" OR "midwife*" OR "midwive*" OR "mental health personnel" OR "medical personnel" OR "medical staff" OR "medical doctor*" OR "medical technician*" OR "medical assistant*" OR "hospital personnel" OR "laboratory personnel" OR "health educator*" OR "pharmacist*" OR "physician*" OR "dentist*" OR "dental*" OR "dietitian*" OR "dietician*" OR "nutritionist*" OR "dietetic*" OR "librarian*" OR "library*" OR "audiolog*" OR "speech therapist*" OR "optometrist*" OR "optician*" OR "laboratory work*" OR "ambulance work*" OR "preventionist*" OR "occupational therap*" OR "physical therap*" OR "physician assistant*" OR "recreational therapist*" OR "social work*" OR "surgeon*" OR "surgical*" OR "case manager*" OR "chiropractor*" OR "chiropractic*" OR "coroner*" OR "pharmacist*" OR "information technology personnel" OR "computer scientist*" OR "IT specialist*" OR "psychiatrist*" OR "psychologist*" OR "psychotherapist*" OR "midwive*" OR "osteopath*" OR "personal trainer*" OR "personal care worker*" OR "health service manager*" OR "health service provider*" OR "life science professional*" OR "biomedical engineer*" OR "medical secretar*" OR "health occupation student*" OR "health occupations student*" OR "medical student*" OR "medical school student*" OR "med school student*" OR "med student*" OR "premedical student*" OR "pre-medical student*" OR "paramedical student*" OR "para-medical student*" OR "healthcare student*" OR "health care student*" OR "pupil nurse*" OR "physical therapy student*" OR "respiratory therapy student*" OR "midwifery student*" OR "midwifery trainee*" OR "chiropractic student*" OR "allied health student*" OR "pharmacy student*" OR "student pharmacist*" OR "pharmaceutical student*" OR "physiotherapy student*" OR "health student*") OR AB ("health personnel" OR "health care personnel" OR "healthcare personnel" OR "health care assistant*" OR "healthcare assistant*" OR "health profession personnel" OR "health professional*" OR "health and hygiene professional*" OR "practice professional*" OR "medicine professional*" OR "health care provider*" OR "healthcare provider*" OR "healthcare worker*" OR "health care

worker*" OR "health worker*" OR "healthcare professional*" OR "health care professional*" OR "health care practitioner*" OR "healthcare practitioner*" OR "paramedical practitioner*" OR "physiotherapist*" OR "clinician*" OR "clinical practitioner*" OR "home health aide*" OR "public health officer*" OR "nurse*" OR "nursing*" OR "midwife*" OR "midwive*" OR "mental health personnel" OR "medical personnel" OR "medical staff" OR "medical doctor*" OR "medical technician*" OR "medical assistant*" OR "hospital personnel" OR "laboratory personnel" OR "health educator*" OR "pharmacist*" OR "physician*" OR "dentist*" OR "dental*" OR "dietitian*" OR "dietician*" OR "nutritionist*" OR "dietetic*" OR "librarian*" OR "library*" OR "audiolog*" OR "speech therapist*" OR "optometrist*" OR "optician*" OR "laboratory work*" OR "ambulance work*" OR "preventionist*" OR "occupational therap*" OR "physical therap*" OR "physician assistant*" OR "recreational therapist*" OR "social work*" OR "surgeon*" OR "surgical*" OR "case manager*" OR "chiropractor*" OR "chiropractic*" OR "coroner*" OR "pharmacist*" OR "information technology personnel" OR "computer scientist*" OR "IT specialist*" OR "psychiatrist*" OR "psychologist*" OR "psychotherapist*" OR "midwive*" OR "osteopath*" OR "personal trainer*" OR "personal care worker*" OR "health service manager*" OR "health service provider*" OR "life science professional*" OR "biomedical engineer*" OR "medical secretar*" OR "health occupation student*" OR "health occupations student*" OR "medical student*" OR "medical school student*" OR "med school student*" OR "med student*" OR "premedical student*" OR "pre-medical student*" OR "paramedical student*" OR "para-medical student*" OR "healthcare student*" OR "health care student*" OR "pupil nurse*" OR "physical therapy student*" OR "respiratory therapy student*" OR "midwifery student*" OR "midwifery trainee*" OR "chiropractic student*" OR "allied health student*" OR "pharmacy student*" OR "student pharmacist*" OR "pharmaceutical student*" OR "physiotherapy student*" OR "health student*"))

SCOPUS (hits, 03/10/2023)

(TITLE-ABS(coronavirus* OR betacoronavirus* OR coronavirinae OR nCoV OR 2019nCoV OR COVID OR COVID19 OR "severe acute respiratory syndrome 2" OR SARS2 OR "cov 2" OR cov2 OR "corona virus*" OR "Wuhan virus") OR AFFIL(nCoV OR 2019nCoV OR COVID OR COVID19 OR “severe acute respiratory syndrome 2” OR SARS2 OR "cov 2" OR cov2 OR "coronavirus*" OR betacoronavirus* OR "corona virus*") OR ((TITLE-ABS(wuhan OR novel OR new OR 19 OR 2019 OR epidem* OR pandem* OR outbreak) OR AFFIL(wuhan OR novel OR new OR 19 OR 2019 OR epidemy OR epidemic* OR pandem* OR outbreak)) AND TITLE-ABS("pneumonia virus*" OR cov OR hcov))) AND (TITLE-ABS("vaccin*")) AND (TITLE-ABS("conspira*" OR "belie*" OR "health-related belief*" OR "antivaccin*" OR "anti-vaccin*" OR "anti-vaxxer*" OR "CTs*" OR "denial*" OR "refusal*" OR "defiance" OR "mistrust" OR "rumor*" OR "plot*" OR "hesitan*" OR "accept*" OR "reject*" OR "willingness" OR "intent*" OR "antiscience*" OR "fake science*" OR "science denial*" OR "science literac*" OR "scientific illiterac*" OR

"scientifically illiterate" OR "misinformation" OR "disinformation" OR "misleading" OR "misinterpretation*" OR "misconception" OR "worldview*" OR "fake news*" OR "false news*" OR "myth*" OR "prejudice*" OR "fake information" OR "information overload" OR "infodemic*" OR "misinfodemic*" OR "infodemiolog*" OR "debunk*" OR "hoax" OR "5G" OR "Bill Gates" OR "ozone" OR "bioweapon*" OR "bio-weapon*" OR "lobby" OR "right-wing" OR "xenophobi*" OR "paranoia*")) AND (TITLE-ABS("health personnel" OR "health care personnel" OR "healthcare personnel" OR "health care assistant*" OR "healthcare assistant*" OR "health profession personnel" OR "health professional*" OR "health and hygiene professional*" OR "practice professional*" OR "medicine professional*" OR "health care provider*" OR "healthcare provider*" OR "healthcare worker*" OR "health care worker*" OR "health worker*" OR "healthcare professional*" OR "health care professional*" OR "health care practitioner*" OR "healthcare practitioner*" OR "paramedical practitioner*" OR "physiotherapist*" OR "clinician*" OR "clinical practitioner*" OR "home health aide*" OR "public health officer*" OR "nurse*" OR "nursing*" OR "midwife*" OR "midwive*" OR "mental health personnel" OR "medical personnel" OR "medical staff" OR "medical doctor*" OR "medical technician*" OR "medical assistant*" OR "hospital personnel" OR "laboratory personnel" OR "health educator*" OR "pharmacist*" OR "physician*" OR "dentist*" OR "dental*" OR "dietitian*" OR "dietician*" OR "nutritionist*" OR "dietetic*" OR "librarian*" OR "library*" OR "audiolog*" OR "speech therapist*" OR "optometrist*" OR "optician*" OR "laboratory work*" OR "ambulance work*" OR "preventionist*" OR "occupational therap*" OR "physical therap*" OR "physician assistant*" OR "recreational therapist*" OR "social work*" OR "surgeon*" OR "surgical*" OR "case manager*" OR "chiropractor*" OR "chiropractic*" OR "coroner*" OR "pharmacist*" OR "information technology personnel" OR "computer scientist*" OR "IT specialist*" OR "psychiatrist*" OR "psychologist*" OR "psychotherapist*" OR "midwive*" OR "osteopath*" OR "personal trainer*" OR "personal care worker*" OR "health service manager*" OR "health service provider*" OR "life science professional*" OR "biomedical engineer*" OR "medical secretar*" OR "health occupation student*" OR "health occupations student*" OR "medical student*" OR "medical school student*" OR "med school student*" OR "med student*" OR "premedical student*" OR "pre-medical student*" OR "paramedical student*" OR "para-medical student*" OR "healthcare student*" OR "health care student*" OR "pupil nurse*" OR "physical therapy student*" OR "respiratory therapy student*" OR "midwifery student*" OR "midwifery trainee*" OR "chiropractic student*" OR "allied health student*" OR "pharmacy student*" OR "student pharmacist*" OR "pharmaceutical student*" OR "physiotherapy student*" OR "health student*")
